# Innate neuroimmunity across aging and neurodegeneration: a perspective from amyloidogenic evolvability

**DOI:** 10.3389/fcell.2024.1430593

**Published:** 2024-07-12

**Authors:** Gilbert Ho, Linh Lam, Tony Tran, Jianshe Wei, Makoto Hashimoto

**Affiliations:** ^1^ PCND Neuroscience Research Institute, Poway, CA, United States; ^2^ Institute for Brain Sciences Research, School of Life Sciences, Henan University, Kaifeng, China; ^3^ Tokyo Metropolitan Institute of Medical Science, Tokyo, Japan

**Keywords:** type 2 immunity, cognition, aging, alarmins, Alzheimer’s disease (AD), β‐amyloid, interleukins, amyloidogenic evolvability

## Abstract

In Alzheimer’s Disease (AD), amyloidogenic proteins (APs), such as β-amyloid (Aβ) and tau, may act as alarmins/damage-associated molecular patterns (DAMPs) to stimulate neuroinflammation and cell death. Indeed, recent evidence suggests that brain-specific type 2 immune networks may be important in modulating amyloidogenicity and brain homeostasis. Central to this, components of innate neuroimmune signaling, particularly type 2 components, assume distinctly specialized roles in regulating immune homeostasis and brain function. Whereas balanced immune surveillance stems from normal type 2 brain immune function, appropriate microglial clearance of aggregated misfolded proteins and neurotrophic and synaptotrophic signaling, aberrant pro-inflammatory activity triggered by alarmins might disrupt this normal immune homeostasis with reduced microglial amyloid clearance, synaptic loss, and ultimately neurodegeneration. Furthermore, since increased inflammation may in turn cause neurodegeneration, it is predicted that AP aggregation and neuroinflammation could synergistically promote even more damage. The reasons for maintaining such adverse biological conditions which have not been weeded out during evolution remain unclear. Here, we discuss these issues from a viewpoint of amyloidogenic evolvability, namely, aEVO, a hypothetic view of an adaptation to environmental stress by AP aggregates. Speculatively, the interaction of AP aggregation and neuroinflammation for aEVO in reproduction, which is evolutionally beneficial, might become a co-activating relationship which promotes AD pathogenesis through antagonistic pleiotropy. If validated, simultaneously suppressing both AP aggregation and specific innate neuroinflammation could greatly increase therapeutic efficacy in AD. Overall, combining a better understanding of innate neuroimmunity in aging and disease with the aEVO hypothesis may help uncover novel mechanism of pathogenesis of AD, leading to improved diagnostics and treatments.

## Introduction

Neurodegenerative disorders traditionally center around misfolded and aggregated protein deposits in brain, such as amyloid neuritic plaques and neurofibrillary tangles in AD, causing neurosynaptic loss (Selkoe and Hardy, 2016), where aging remains the strongest risk factor for development. Accumulated evidence highlights brain immune networks as important not only for maintaining normal brain function during aging, but also in the abnormal pathogenesis of Alzheimer’s Disease (AD) and related neurodegeneration. Indeed, the notion of the brain as an “immunologically-privileged” organ has been dispelled, paving the way toward a revised understanding of the functioning of the innate neuroimmune system in brain. Critical to modulating brain innate immunity are microglia/macrophages along with astrocytes, with actions both supportive and damaging to neurons/synapses depending on the nature and combination of cytokine/chemokine signals. Widely known, brain microglia and astrocytes are linked in a spectrum of activities ranging from pro-inflammatory to anti-inflammatory, which, when “dialed-in” for homeostatic balance, provide trophic support for neuronal function, modulating synaptic activity, reorganizing neuronal circuits, and maintaining the blood brain barrier (Matejuk and Ransohoff, 2020). In AD, another major risk factor, triggering receptor expressed on myeloid cells 2 (TREM2), increases microglial phagocytosis of Aβ, and also exerts anti-inflammatory effect on Toll-like receptor (TLR) activation (Guerreiro et al., 2013; Jonsson et al., 2013; Liu et al., 2020). Yet, loss of function TREM2 variants markedly increase late-onset AD risk, perhaps through promoting inflammation (Gratuze et al., 2018). Thus, a sustained low-level chronic neuroinflammatory state may underlie changes across normal aging into neurodegeneration.

Driving inflammation during aging and into neurodegenerative disorders, alarmins serve as essential signals of tissue damage or insult. On the one hand, they activate many pro-inflammatory signaling networks, including the ubiquitous innate pro-inflammatory mediators, interleukin (IL)-1β and IL-6, which, along with tumor necrosis factor (TNF)-α, which are known to increase amyloidogenic pathology in AD (Erta et al., 2012; Brough and Denes, 2015; Su et al., 2016; Liu and Quan, 2018; Gonzalez Caldito, 2023). In response, type 2 immunity may be considered counter-regulatory not only against type 1 T helper (Th1), but also against other pro-inflammatory responses to suppress excess brain inflammation. Yet, what remains incompletely understood is how type 2 immunity interacts with brain aging leading to Alzheimer and neurodegenerative pathology. A further exploration of type 2 responses reveals that chronic low level inflammation might develop from chronic disequilibrium between type 2 signaling and pro-inflammatory factors, but beyond simply countering inflammation, brain type 2 immunity has emerged as a key player in precision monitoring and coordination of astrocytic and microglial actions in support of neurosynaptic homeostasis (Mamuladze and Kipnis, 2023). During the progress of aging, these systems undergo gradual functional dysregulation, leading to chronic brain inflammation, predisposing to AD and other neurodegenerative pathology. Understanding such alterations in these systems offers unique opportunities for identifying potential molecular drug targets for neurodegeneration.

Envisioning possibilities, abnormal brain innate neuroimmunity might also influence another important putative physiological function related to the aggregation of amyloidogenic proteins in AD. Provided that evolution generally favors processes and characteristics optimized toward beneficial biological outcomes, the question arises as to why a chronic abnormal innate immune state develops during aging and is not screened out during evolution. Since most of our concept of AP aggregation in relation to AD is from the pathological perspective, perhaps a better understanding of the precise physiological functions of APs would prove more insightful. Thus, we recently proposed an AP aggregate-based adaptation to environmental and biological stressors, namely, amyloidogenic evolvability (aEVO), that might be physiologically significant for aging and AD (Hashimoto et al., 2018a; 2018b). Because neuroinflammation is a major biological stressor, we also explore the interaction of AP aggregation with neuroinflammation as relevant to the concept of aEVO. In this context, we will discuss how Aβ aggregation might interact with inflammatory networks to promote aEVO during reproductive life, and eventually lead to AD and neurodegenerative disorders through antagonistic pleiotropy during aging. Accordingly, we propose that targeted and selective co-suppression of AP aggregation and neuroinflammation could significantly improve therapeutic efficacy in AD.

### The complex spectrum of innate neuroimmunity

**FIGURE 1:**
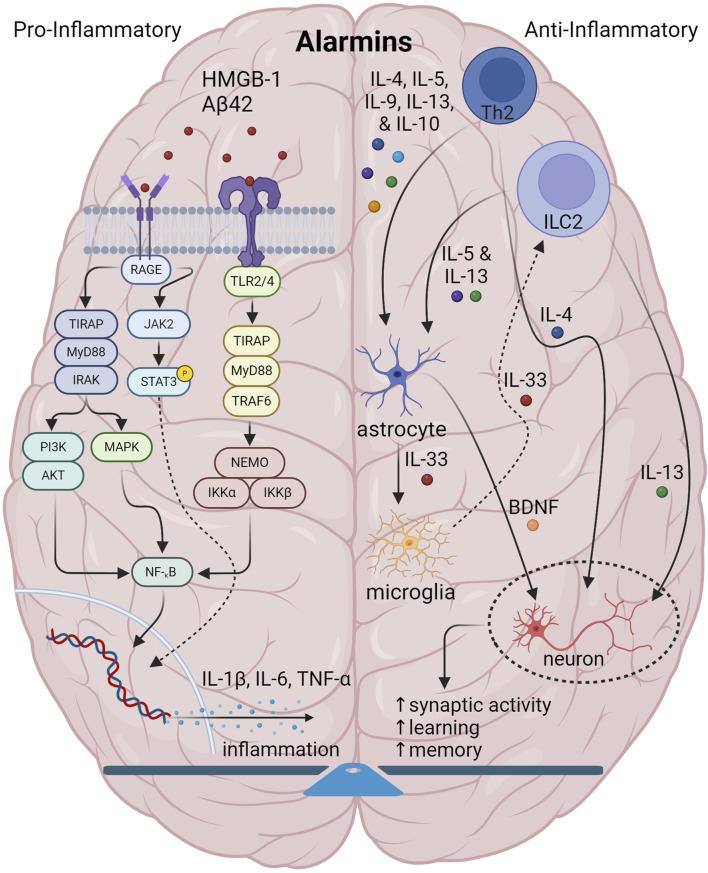
Illustration showing the interplay among components of normal brain innate immunity. In normal brain innate immunity, there is a distinct equilibrium of pro-inflammatory/Th1 and anti-inflammatory activities. On the one hand, pro-inflammatory alarmins signal through TLR2/4 and RAGE axes to generate pro-inflammatory cytokines, IL-1, IL-6, TNF-α and others which have a pleiotropic effect on multiple CNS cells. On the other side, type2 immunity initiated by Th2 and DCs and gated through ILC2s, facilitate the release of canonical mediators, IL-4, IL-5, IL-9, IL-10 and IL-13 to not only counter inflammation, but also to coordinate and regulate CNS function. Of specific interest, IL-4 and IL-13 specifically target neurons and synapses to promote plasticity, memory and learning. Also, IL-33 is important in promoting microglia phagocytosis. Lastly, IL-4 targets GABA neurons and astrocytes to generate BDNF to support cognition.

During innate immunity, illustrated in Figure 1, pro-inflammatory alarmins such as high-mobility group box 1 (HMGB1) and others activate PRRs such as TLR2 and -4, and receptor for glycation end-products (RAGE) to trigger other cascading signaling. Whereas RAGE signals primary through Ras/extracellular signal regulated kinase (Erk) 1/2 and p38 as well as through calcium calmodulin-dependent protein kinase kinase-β-AMP-activated protein kinase (CaMKKbeta), TLR2/4 signaling activates adapter proteins inducing nuclear factor kappa-light-chain-enhancer of activated B cells (NF-κB) to eventually trigger the macro-assembly and auto-activation of the NLRP3 inflammasome with apoptosis-associated speck like protein containing a CARD (ASC) and pro-caspase-1 (Yang et al., 2017). Eventually this causes release of pro-inflammatory chemokines and cytokines such as IL-1β and IL-6, which, along with TNF-α, which are known to drive amyloidogenic pathology in AD (Su et al., 2016).

In contrast, type 2 immunity or Th2 immunity, an adaptive response to parasites (Gause et al., 2013) and atopic allergens (Galli et al., 2008), at epithelial barriers and parenchymal borders (Akdis, 2021), is considered in some contexts to be anti-inflammatory, to balance out the action of pro-inflammatory molecules, and, as such, is critical in tissue repair and regeneration (Gieseck et al., 2018). Triggered by alarmins, type 2 response is largely defined by the activity of the canonical interleukins, IL-4, IL-5, IL-9, IL-10, and IL-13. In the central nervous system (CNS), type 2 responses play very specialized roles in regulating the interplay among glial function and neurosynaptic activity to maintain normal homeostatic balance and connectivity (Mamuladze and Kipnis, 2023). Conceptually, such signaling is initiated by a tissue disturbance from external stimuli ranging from bacteria, viruses, parasites, allergens and others, which sensitize dendritic cells (DCs) and T helper 2 (Th2) cells, along with eosinophils, basophils and macrophages to generate the main type 2 interleukins. Now recognized, cellular responses are also mediated by innate lymphoid type 2 cells (ILC2), are now recognized as equally important as Th2 cells and DCs. A number of alarmins can stimulate ILC2s through receptor signaling including IL-33, which activates ST2 receptor, IL-25, which activates IL-17RA/IL-17RB complex, and thymic stromal lymphopoietin (TSLP) through the TSLP receptor complex, with the list ever-expanding. Combinations of stimulatory, co-stimulatory and inhibitory molecules also specify the net response. For instance, at least in pulmonary tissue, co-stimulatory activation through TNF-α and stem cell factor (SCF) binding c-Kit greatly enhance IL-33 and IL-25 activity. In contrast, IL-10, IL-27, and transforming growth factor-β (TGF-β) act as inhibitory factors, along with non-cytokine mediation by lipids, i.e., prostaglandins, neuropeptides, and hormones such as acetylcholine, vasoactive intestinal peptide, and calcitonin-gene related peptide (Thio and Chang, 2023). With a pathogen or allergic stimulus, ILC2 and DC crosstalk also enables the recruitment of Th2 signaling and eventual expression of IL-4, IL-5 and IL-13 to affect appropriate inflammation and tissue repair (Wynn, 2015). Dysregulated type 2 immune activity has been implicated in various chronic disease states ranging from asthma and allergy, dermatitis to inflammatory bowel disorders, also due to an aberrant chronic inflammatory state (Lloyd and Snelgrove, 2018).

Yet, how type 2 immune responses in the context of brain innate immunity are altered across aging and in Alzheimer and neurodegenerative pathology remains less well-understood, but evidence now suggests that, similar to other atopic conditions, a chronic low-level inflammation might develop from dysregulation of type 2 signaling, associated with disrupted cross-talk between glial cells and neurons and neurosynaptic failure. In the brain, alarmins and type 2 interleukins are thus critical in modulating astrocytes and microglia to support neurosynaptic homeostasis, which is progressively degraded during aging and in some cases transforming to neurodegeneration.

### Alarmins drive innate brain immunity in aging and AD

Driving inflammation during aging and into neurodegenerative disorders, alarmins, also designated as danger-/damage-associated molecular patterns (DAMPs), serve as general molecular danger signals in innate immunity indicating cellular or tissue injury (Yang et al., 2017). As a diverse and ever-expanding group of proteins and peptides from many cellular sources, they respond to various biological threats by activating chemotactic and pattern recognition receptors (PRRs) to initiate diverse tissue-dependent host responses, including defending against microbes, regulating gene expression, maintaining cellular homeostasis, wound healing, immune/autoimmune functions and even oncogenesis. Facilitating this, alarmins attract and activate leukocytes and mast cells to produce histamines, prostaglandins, chemokines and cytokines, among other immune molecules (Yang et al., 2017). As mentioned, pro-inflammatory alarmins commonly signal through TLR2/4 and RAGE whereas type 2 alarmins, IL-33, IL-25 and TSLP, which originate from brain hematopoietic, stromal, and parenchymal sources, signal through their respective cognate receptors (Stanbery et al., 2022). Yet, beyond mere uni-directional activation, a critical feature of these molecules might be that they are also generated by astrocytes and oligodendrocytes, feeding back and causing auto-amplification and rapid spread of inflammatory signals across tissue areas and networks. Indeed, in a mouse model of virally-damaged lung tissue, HMGB1/RAGE signaling activated ILC2s to produce IL-13, generating an HMGB1/IL-13 auto-amplification loop, whereby IL-13 indirectly damages airway smooth muscle *via* elaborated TGF-β (Loh et al., 2020). Similarly in the brain, pathogen-associated molecular patterns induced astrocytic production of IL-33 and IL-13 in cell culture, and IL-33 directly induces immune gene expression in various CNS glia, which strongly suggests that immune molecules can form both autocrine and paracrine auto-amplification loops between astrocytes and various other CNS immune cells (Hudson et al., 2008; Rao et al., 2022). Thus, in the brain, astrocytic IL-33, induced by pro-inflammatory molecules, could conceivably form an auto-amplification loop, progressively increasing both IL-33 and pro-inflammatory cytokine production and neuroinflammation. In parallel, astrocytic IL-33 could also activate microglia to produce inflammatory molecules such as IL-1β, TNF-α, and many others, which loop backward to further activate microglia, generating another pro-inflammatory auto-amplification loop. Collectively, such rapid amplification of immune signals may partly contribute to chronic low-grade neuroinflammation during aging (Rao et al., 2022), and aside from promoting inflammation, such loops may also functionally dysregulate the physiologic activity of brain type 2 immune molecules during both aging and in AD and neurodegeneration.

Remarkably, alarmins also appear to bridge innate immunity with cellular senescence and neurodegeneration. Emerging alarmins in cognitive and mental disorders including HMBG1, S100, IL-33 and its soluble receptor, soluble ST2, while among type 2 alarmins, less is known about the roles of TSLP and IL-25, but IL-25 could be important in maintaining blood-brain barrier (BBB) integrity (Sonobe et al., 2009; Yuan et al., 2023). Undoubtedly, HMGB1, a highly-conserved and ubiquitous non-histone nucleoprotein, plays a pivotal role in abnormal chronic inflammation in aging and AD. Composed of two DNA-binding domains and an acidic end domain, it normally confers chromatin stability, but is actively translocated and secreted following various stimuli such as cell damage, toxins, or hypoxia (Mao et al., 2022). In AD brain, HMGB1 is significantly increased and co-localizes with amyloid plaques (Xu et al., 2024), and inhibits microglial Aβ42 uptake, increasing Aβ42 accumulation and amyloidogenicity (Takata et al., 2003). Recently, HMGB1 was designated an inflammatory marker of cellular senescence and perhaps an important driver of tau pathology in AD (Gaikwad et al., 2021; Gaikwad and Kayed, 2022). Similarly, the S100 family of EF-hand calcium proteins also produce inflammation through RAGE activation and are implicated in Alzheimer pathology (Venegas and Heneka, 2017; Cristóvão and Gomes, 2019).

A member of the large IL-1 gene family, IL-33 importantly coordinates networking activity between the CNS and type 2 immunity. A pleiotropic type 2 alarmin, the molecule remains rather resilient in the brain during aging and may be directly more important in maintaining neurosynaptic function during senescence. Actively secreted by stromal and barrier tissues in other organs, it is also released in the brain by astrocytes and oligodendrocytes to engage neighboring astrocytes and microglia expressing the cognate ST2 receptor (Fu et al., 2016; Liew et al., 2016). Since during brain development, excessive excitatory synapses cause abnormally overactive brain circuitry, a primary function of IL-33 is to signal microglia to activate synaptic engulfment and pruning, reducing excess or redundant synapses to ensure normal neural circuit refinement (Vainchtein et al., 2018). Of note, this process of microglial synaptic maintenance continues throughout the lifespan, which might implicate IL-33, interacting with secreted phosphoprotein1, in synapse loss in AD (Terry et al., 1991; De Schepper et al., 2023). Overall, this process is particularly relevant to learning and memory, since it modulates glial-synaptic activity to maintain connectivity in select hippocampal circuits (Wang et al., 2021).

### Type 2 immunity supports neurosynaptic function in memory and learning

With regard to neuronal function in aging and Alzheimer’s disease, evidence points to a pivotal role of type 2 immune signaling in modulating the activity of microglia and astrocytes in brain for proper neurosynaptic function. Chronic dysregulation of these signaling networks, on the other hand, may lead to abnormal neuronal function, altered synaptic plasticity and accelerate amyloidogenic pathology such as deposition of amyloid, tau and alpha-synuclein. Reflecting the complexity of brain structure and organization, brain type 2 immune components play remarkably specialized roles to support normal neuronal activity linked to learning and memory. Indeed, an excellent scheme outlining brain type 2 neuroimmunity has recently been described (Mamuladze and Kipnis, 2023).

Highlighting key aspects of type 2 neuroimmunity, the leptomeninges and their lymphatic vessels, dura mater, and choroid plexus are immune-rich sources for ILC2s, Th2, basophils, and mast cells all of which detect brain-derived antigens washed here by cerebrospinal fluid and also by cervical and lymphatic flow (Gadani et al., 2017). The elaboration of IL-4, IL-5, IL-9 and IL-13 into the cerebral milieu activates glia to produce neurotrophic factors, which along with IL-4 and IL-13, enhance neurosynaptic function and learning and memory (Mamuladze and Kipnis, 2023). Alarmins, primarily IL-33, are also produced to activate microglial phagocytosis and also to feed backward to activate ILCs, Th2, mast cells and basophils to promote the type 2 response.

IL-4 induces signaling by binding with either type 1 receptors comprised of IL-4Rα and a gamma chain, or type 2 receptors containing both the IL-4Rα and the IL-13Rα1 subunits. IL-4Rα receptors are expressed on GABAergic neurons and in astrocytes which produce brain-derived neurotrophic factor (BDNF) for learning and memory (Gadani et al., 2012). As observed in hematopoietic cells, type 1 receptors likely signal through Janus kinase (JAK) 1/3—signal transducer and activator of transcription (STAT) six–insulin receptor substrate two and mitogen-activated protein kinase (MAPK), whereas type 2 receptors signal through JAK1 and tyrosine kinase (TYK) 2, causing STAT6 phosphorylation, dimerization and nuclear translocation (Heller and Dasgupta, 2012). Similarly, IL-5 effects are mediated by its cognate receptor, IL-5R, which also generally signals through Ras-MAPK and JAK-STAT system, although Lyn, Syk and JAK2 tyrosine kinases are activated for eosinophil survival, having unclear significance for the brain (Adachi and Alam, 1998). Speculatively, given the similarity to IL-4, it may also promote astrocyte BDNF production. Less is known about IL-9 and IL-10 role in neural function, but they are believed to be anti-apoptotic and/or neuroprotective (Noelle and Nowak, 2010; Ding et al., 2015; Porro et al., 2020).

Moreover, IL-13 and its receptor IL-13Rα1 are neurosynaptic proteins, and neurons treated with IL-13 are less vulnerable to excitotoxic cell death (Li et al., 2023). IL-13, mostly localized to pre-synaptic membrane, signals through a type-I receptor, mostly found post-synaptically, comprising an IL-13Ra1 subunit heterodimer with IL-4 receptor alpha chain. Present in mouse, rat and human neurons, synaptic IL-13 receptor binding to IL-13Ra1 induces phosphorylation of glutamatergic N-methyl-D-aspartate (NMDA), α-amino-3-hydroxy-5-methyl-4-isoxazolepropionic acid (AMPA) receptors, Ca_2_^+^/calmodulin-dependent protein kinase II (CaMKII) and protein kinase A (PKA), leading to JAK2/TYK2-dependent phosphorylation of the transcription factors STAT3 and STAT6 and CREB-mediated transcription of multiple immediate early genes to augment synaptic activity and plasticity (Goenka and Kaplan, 2011; Li et al., 2023). Also, the decoy receptor, IL-13Rα2, not only binds IL-13, but also downregulates IL-4 signaling through inhibiting IL-4Rα dimerization with its second subunit (McCormick and Heller, 2015).

Thus, without a doubt, IL-4, IL-5, IL-13 and IL-33 appears disproportionately targeted toward memory and learning regulation. With regard to behavior, IL-13 knockout mice demonstrated deficient learning and memory (Brombacher et al., 2017). Also, mice depleted of meningeal IL-4-producing T-cells, as well as IL-4 knockout mice both show impairment in the Morris water maze learning and memory paradigm (Derecki et al., 2010). Also, T-cell-depleted SCID mice with reduced IL-4 have disrupted contextual fear memory (Herz et al., 2021). Consistently, IL-4 binding IL-4Rα also directly facilitates memory and synapse function in GABAergic neurons. Also, IL-5 administration in transgenic AD mice similarly improves spatial learning and recognition ability (Fung et al., 2021).

Of significance, the direct modulation of glutamatergic synaptic activity by IL-13 to maintain learning and memory, and its potential pathological disruption, is particularly reminiscent of a key neuropathological feature in AD brain, namely, the widespread loss of synapses and brain connectivity (Terry et al., 1991) suggesting a mechanistic connection. Importantly, IL-4 and IL-13 both appears to stimulate astrocytes to produce several trophic factors, most prominently brain-derived neurotrophic factor (BDNF), a key molecule in promoting learning and memory (Brombacher et al., 2017). Of interest, IL-13 induces rapid phosphorylation of many important synaptic proteins other than glutamate receptors, such as alpha-synuclein, suggesting the possibility that IL-13 deficiency may also play a role in Parkinson’s disease. A more detailed summary of the pro-inflammatory and anti-inflammatory type 2 innate immunity effectors is provided in Table 1.

**TABLE 1:** Innate immunity effectors in the brain and cognition. Summarizes the principal pro-inflammatory and type 2 anti-inflammatory molecular effectors of innate immunity and their expression characteristics, signaling axes, and relevance to brain function and cognition where appropriate. A, astrocytes; BBB, blood-brain barrier; DAP12, DNAX-activation protein 12; GPCR, G-protein-coupled receptor; hCSIF, human cytokine synthesis inhibitory factor; HPA, Hypothalamo-pituitary-adrenal axis; IL-1RAcP, IL-1 receptor accessory protein; Leuk, leukocytes; Mϕ, macrophages; M, Microglia; N, neurons; O, oligodendrocytes; TBI, traumatic brain injury; TLR, Toll-like receptor; RAGE, Receptor for advanced glycation end-products.

	Effector	Cognate Receptor(s)	Targets	Major signaling axis	CNS Function(s)	CNS disease states	References
Pro-inflammatory	IL-1α, IL-1β	Type I IL-1 receptor (IL-1RI) IL-1RII decoy receptor	Diverse; hippocampal dentate gyrus and pyramidal cells	MyD88 - NFκB and MAPK—AP-1—inflammasome	Learning/Memory, HPA axis, Injury response	AD, PD, MS, TBI, Stroke, Epilepsy, Mood Disorders, Autism	Brough and Denes (2015) Su et al. (2016) Liu and Quan (2018)
	IL-6	IL6R IL-6Rα binding chain (CD126) and β-receptor gp130 (CD130)	Classical signaling: M Trans-signaling: M, A, N, O	JAK/STAT—MAPK – PI3K—IRS	Acute injury response, neuronal homeostasis	AD, PD, MS, Huntington’s, Stroke	Erta et al. (2012)
	TNF-α	TNF-α receptor 1 (TNFR1) TNF-α receptor 2 (TNFR2)	TNFR1: N, M, A TNFR2: N	TNFR1: NFκB—MAPK or caspase 8; TNFR2: IκB kinase – NFκB—MAPK	Brain homeostasis, synaptic plasticity, Oligodendrocyte functions (TNFR2 only)	MS, Autoimmune disorders	Gonzalez Caldito (2023)
	HMGB1	TLR2/4, RAGE	M, A	MyD88 - NFκB and MAPK—AP-1—inflammasome	Chromatin stability, innate inflammatory alarmin	AD, PD, Stroke, TBI, Epilepsy, Autism, MS, ALS, Depression	Mao et al. (2022) Xu et al. (2024)
	S100	TLR4, RAGE, GPCR, others	N, M, A depending on S100 subtype	NFκB and multiple others	Alarmin, cell growth, calcium homeostasis, *etc.*	AD	Vengas and Heneka (2017) Cristóvão and Gomes (2019)
Anti-inflammatory	IL-4	IL-13Rα1/IL-4Rα heterodimer	N (GABAergic), A	JAK/STAT6 and IRS-1/2—PI3K—Akt	BDNF for learning and memory, synaptic function	AD, MS, GBM	Gadani et al. (2012)
	IL-9	IL-9R (IL-9Rα + γ-chain)	A, M, O (and progenitors)	JAK/STAT	Neuroprotective, anti-apoptotic	MS and AD	Noelle and Nowak (2010) Ding et al. (2015)
	IL-10 (hCSIF)	IL-10R (IL-10Rα + IL-10Rβ)	Leuk, Mϕ, M, A, N, O	↓ inflam. by JAK/STAT, ↑ growth by PI3K/Akt	Neuroprotective	AD, MS, TBI, ALS	Porro et al. (2020)
	IL-13	IL-13Rα1/IL-4Rα heterodimer	N, A	JAK/STAT6, ?MAPK	Synaptic plasticity, learning/memory, microglial phagocytosis	AD, TBI	Brombacher et al. (2017) Li et al. (2023)
	IL-25	IL-17RA/IL-17RB heterodimer complex	Receptor expressed in mouse brain	TRAF adaptors – MAPK—NFκB—JAK/STAT	BBB integrity	MS, Autoimmune Disorders, ?AD, ?PD	Sonobe et al. (2009) Stanbery et al. (2022) Yuan et al. (2023)
	IL-33	ST2(IL1RL1)/IL-1RAcP heterodimer	M, A, O	MyD88—MAPK—NFκB—AP-1—CREB	Pleiotropic, synaptic engulfment, type 2 alarmin	AD, PD, MS, TBI, Stroke	Saresella et al. (2020) Stanbery et al. (2022)

## Aging dysregulates brain type 2 interleukins

Early evidence indicates that type 2 neuroimmunity is altered by senescence, where studies have focused on functional changes in IL-4. Importantly, hippocampal IL-4 concentrations are significantly reduced in an aging rat model resulting in attenuated long-term potentiation (LTP) and synaptic plasticity (Nolan et al., 2005). As IL-4 normally suppresses IL-1β signaling-related reduction in LTP by down-regulating hippocampal type I interleukin-1 receptor (IL-1RI) expression, IL-4 restoration in rats reverses age-associated reduction in hippocampal LTP through increased JAK1 and STAT6 phosphorylation (Nolan et al., 2005). Beyond aging, one might speculate that age-associated loss of IL-4 signaling might increase neuronal susceptibility to other degenerative insults, which could be rescued by increasing IL-4. Yet, surprisingly more complex, levels of interferon (IFN)-γ decrease and IL-4 also increase in the choroid plexus (CP) epithelium during aging, where IL-4 instead becomes neurotoxic by releasing the chemokine, C-C motif chemokine (CCL) 11 or eotaxin-1. A chemotactic factor for eosinophils in asthma and allergy, CCL11 signals through G-protein coupled receptors (Baruch et al., 2013), and mitigates excess IL-4 signaling in CP epithelium and meninges, but adversely induces excitotoxic neuronal death by attracting microglia to generate reactive oxygen species through nicotinamide adenine dinucleotide phosphate-oxidase 1 (NOX1) (Parajuli et al., 2015). Indeed, IL-4 alterations during senescence are complicated and may also be regionally-dependent in brain, so specific targeting of IL-4 to vulnerable areas may be required. Simultaneously, the astrocytic expression of the alarmin, IL-33, a member of the IL-1 cytokine family, becomes elevated during aging (Carlock et al., 2017), and IL-33 knockout even at middle age in mice leads to severe oxidative damage, defective repair of DNA double-strand breaks, neurosynaptic loss, and tau pathology similar to AD (Lou, 2021). Related to IL-33, activated ILC2 from choroid plexus, being resistant to senescent changes, when injected intracerebroventricularly, also suppressed aging neuroinflammation and cognitive decline in aged mice, presumably through expression of neuroprotective IL-5 (Fung et al., 2020).

### Type 2 immunity is altered in AD neurodegeneration

Consistently, multiple AD pathologies are indeed exacerbated by dysregulated type 2 activity, which might be important mechanistically in AD pathogenesis. Central to the amyloid cascade hypothesis, accumulated Aβ in the cortex leads to fibrilization and neurotoxicity (Selkoe and Hardy, 2016). Accordingly, IL-33 readily promotes Aβ phagocytosis and clearance by microglia, a major route of elimination of excess brain Aβ, and since amyloid precursor protein (APP)/presenilin-1 (PS1) mice transgenic for β-amyloid overexpression show cognitive and behavioral deficits, IL-33 therapeutic injection reduced both amyloid burden and rescued contextual memory deficits (Fu et al., 2016). In patients, serum and CSF IL-33 and IL-10 levels were significantly reduced in AD and mild cognitive impairment (MCI), whereas IL-1β levels were increased. LPS- and Aβ42-treated monocytes from MCI patients and controls downregulated IL-1β expression and also increased IL-10 secretion in control monocytes, whereas AD monocyte expression of IL-33 and IL-10 remained unchanged (Saresella et al., 2020). Correspondingly, levels of soluble ST2, an IL-33 decoy receptor, are significantly increased in MCI serum (Fu et al., 2016; Saresella et al., 2020) and also in AD serum (Saresella et al., 2020), suggesting that normalizing IL-33 may be a relevant strategy in AD, yet must occur very early in disease course to be effective. Interconnected with IL-33, significantly attenuated and functionally defective ILC2s were found in triple transgenic AD mice (3xTg-AD), overexpressing amyloid, tau and presenilin-1, which also produced abnormally decreased amounts of protective IL-5 and developed pro-inflammatory gene expression of granzyme A, cathepsin A, and cathepsin H (Fung et al., 2021).

Remarkably, alterations in IL-4, IL-10 and IL-13 might also be associated with AD pathological features. Although total IL-4 plasma levels were unchanged in MCI and AD relative to controls, IL-4 levels in both groups were associated with hippocampal volumes in the subiculum and presubiculum as assessed by volumetric magnetic resonance imaging, again suggesting a neuroprotective effect on vulnerable regionally-specific hippocampal neurons (Boccardi et al., 2019). Speculatively, a functional compromise in IL-4 protective effect on hippocampal circuits in AD could lead to a compensatory increase in levels, and indeed, peripheral IL-4 and IL-10 were both highly elevated only in subsets of AD patients with more rapid cognitive decline (Leung et al., 2013). Although limited data exists regarding IL-9 alteration with aging and neurodegeneration, a curious finding was reported by Wharton et al. (2019), indicating that elevated CSF IL-9 levels were only associated with African American AD patients, and consistently in two separate large cohorts, post-mortem brain IL-9-related molecular markers were also present only in African-American AD subjects.

A recent postmortem study also identified significantly elevated IL-13 levels, as well as IL-1α and IFN-γ and granulocyte-macrophage colony-stimulating factor in mid-temporal AD cortex. Moreover, increased IL-10 and IL-13 were significantly associated with moderate to severe neurofibrillary tangle pathology in brain, but not amyloid plaques or Lewy bodies, indicating perhaps that IL-10 and IL-13 might contribute more to tau than amyloid pathology. Also, IL-10 and IL-13 alterations in brain appear specific to AD, and not Parkinson’s disease (PD) or Dementia with Lewy Bodies, which have separate immunologic signatures (Chai et al., 2023), and could prove interesting as future differential biomarkers for these conditions.

### The aggregation of APs enhances neuroinflammation

Given the present understanding of innate immunity, neuroinflammation may be one major consequences of the aggregation of APs, especially pro-inflammatory through the TLR2/4 and RAGE signaling axes. Supporting this, Aβ 25–35 treatment upregulated HMGB1 in cultured hippocampal neurons, in which TLR4/NF-κB and RAGE are also upregulated (Nan et al., 2019). Also, fibrillar Aβ peptides are shown to activate microglia *via* TLR2 under cell-based conditions (Jana et al., 2008). Similarly, CD14 and TLR4 in addition to TLR2 are required for fibrillar Aβ-stimulated microglial activation and the elaboration of pro-inflammatory cytokines (Reed-Geaghan et al., 2009). In line with the above, Aβ aggregates sensitized TLR4 signaling causing long-term potentiation defects and rat neuronal cell death (Hughes et al., 2020), and TLR4-dependent upregulation of cytokines was observed in transgenic mice model of AD (Jin et al., 2008). Alternatively, Aβ42 also signals through RAGE/NF-κB which promotes neuronal differentiation in hippocampal neural progenitor cells from TgCRND8 transgenic mice (Meneghini et al., 2013). Yet, on balance, Szczepanik et al. reported that Aβ-treated cultured human THP-1 monocytes actively produced IL-4, IL-10 and IL-13 in a time- and dose-dependent manner to differentially regulate cultured microglial responses to Aβ, suppressing pro-inflammatory IL-6, and depending on tissue type, IL-1α, IL-1 β, and TNF-α (Szczepanik, 2001) Meanwhile, inflammatory protein dot blot array demonstrated that Aβ42-treated SH-SY5Y cells, α-synuclein overexpression, or combination of both, differentially expressed type 2 cytokines and chemokines, including chemokine C-C motif ligand (CCL) 2, IL-13 and IL-4 (Keskin et al., 2023). These may represent a simultaneous compensatory response of brain type 2 immunity to counter aberrant inflammation in AD pathogenesis, which if deficient during aging or disease, could further accelerate Aβ-induced pro-inflammatory activity.

Among other findings, provided that patients carrying apolipoprotein (ApoE) ε4 allele(s) linked to increased sporadic AD risk, enhanced Aβ aggregation, neurodegeneration, and neuroinflammation (Parhizkar and Holtzman, 2022), Ulrich and colleagues found that in APPPS1ΔE9 and APPPS1-21 transgenic mice, fibrillar Aβ deposition and also inflammatory microgliosis was dramatically reduced in the absence of ApoE, indicating that this may be an important inflammatory mediator of Aβ (Ulrich et al., 2018). Also, patients harboring familial AD mutations, such as amyloid precursor protein and presenilin (PS)-1/2, show increased Aβ aggregation rates (Hatami et al., 2017), and mutant PS2 transgenic mice displayed microglial abnormalities, increased TREM2 and IL-6 expression in culture, thus promoting inflammation (S. Fung et al., 2020). Importantly, small aggregates of Aβ fibrils, such as oligomers and protofibrils, that exhibit potent cellular and neuronal toxicity, also cause enhanced inflammation. Indeed, the contact system which can promote coagulation and inflammation, was preferentially activated by protofibrils of Aβ (Zamolodchikov et al., 2015; Singh et al., 2020). Aβ protofibrils bind to coagulation factor XII and high molecular weight kininogen and accelerate the activation of the system resulting in vascular and inflammatory pathologies (Yamamoto-Imoto et al., 2018). Curiously, it was recently shown that dementia-related angiopathy could be related to a disruption of vascular endothelial-cadherin junctions as induced by exposure to nanoparticulates of Aβ oligomers and seeds, which appears to be an earlier event prior to pro-inflammatory and pro-oxidative stressors to endothelial leakiness (Li et al., 2024). Collectively, we propose that Aβ, in its multiple forms, may also be classified as a damage associated molecular pattern (DAMP) or alarmin, which by definition, triggers the innate immunity in the brain, leading to both neuroinflammation and compensatory type 2 responses in the AD brain (Yang et al., 2017).

### Neuroinflammation conversely induces AP aggregation

Conversely, recent investigations suggest that neuroinflammation might also increase the aggregation of APs. With this in mind, various transgenic approaches in AD animal models and also cell-based paradigms have provided evidence that enhancing inflammation is linked with increased Aβ generation and aggregation. For instance, elevated HMGB1 co-localizes with neuritic plaques in AD brain and inhibits microglial Aβ42 removal which would promote amyloidogenesis (Takata et al., 2004). Also, pro-inflammatory RAGE signaling is well-known to cause amyloidogenesis, tau hyperphosphorylation and other AD pathologies (Cai et al., 2016), and genetic deletion of inflammatory genes, such as TNF-α, and inducible nitric oxide synthetase, ameliorated neuropathological features in various types of AD transgenic mice (Kummer et al., 2011; Paouri et al., 2017). Of significance in patients, AD risk is significantly elevated through compromised microglial function and reduced Aβ clearance related to rare mutations in the TREM2 gene (Guerreiro et al., 2013; Jonsson et al., 2013), leading to Aβ accumulation.

Amongst type 2 anti-inflammatory components, IL-33, whose function might be reduced in AD due to increased soluble ST2 receptor decoy levels, when administered to transgenic Aβ AD mice, promotes Aβ microglial clearance, reducing soluble Aβ and amyloid plaque pathology (Fu et al., 2016). Similarly, in transgenic Aβ/PS1 bigenic mice, overexpression of IL-4 markedly reduces glial pathology and Aβ oligomerization, while improving cognition (Kiyota et al., 2010). Lastly, IL-4-treated microglial cultures and APP23 dual mutant (K670N/M671L) mice induced CD36 scavenger receptor, neprilysin Aβ-degrading enzyme and insulin-degrading enzyme (IDE), all of which promote microglial Aβ removal and breakdown, reducing accumulated and aggregated brain Aβ (Shimizu et al., 2008). Therefore, considering that they exert an anti-aggregative effect on APs by promoting degradation or microglial removal, one implication is that in aging and AD, IL-33 andand IL-4 activity could be compromised, instead leading to increased AP aggregation.

### Bidirectional regulation of AP aggregation and neuroinflammation influences aEVO

Increasingly apparent, the processes of AP overexpression/aggregation and neuroinflammation likely exist in a balanced bidirectional relationship, promoting normal amyloid function, neurosynaptic connectivity, and healthy cognition. Intrinsic in this, the delicate balance between pro-inflammatory innate immunity and type 2 actions allows for normal physiologic amyloidogenesis, while as discussed, during aging and neurodegeneration, these relationships are perturbed. On the evolutionary scale, these phenomena likely emerged in the human brain only fairly recently. Conceptually, based on natural selection, pathogenic phenomenon such as amyloidogenicity in AD might be linked to certain beneficial physiological functions of amyloid production during the human reproductive stage, otherwise it would have been eliminated during evolution.

**FIGURE 2:**
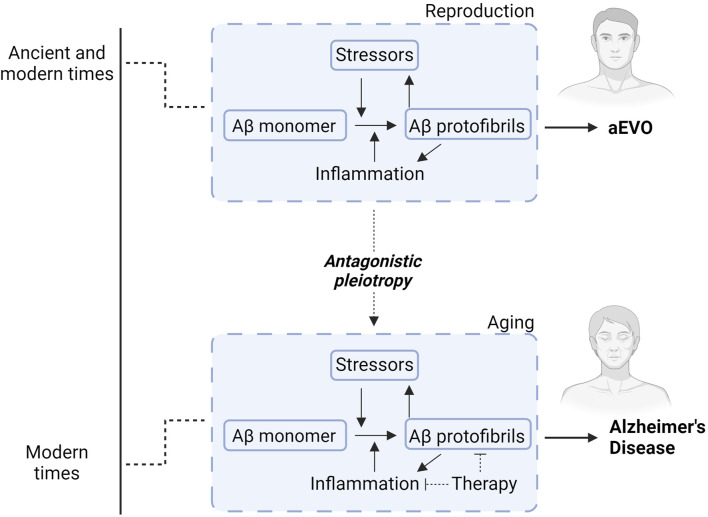
Diagram illustrating how Aβ aggregation and neuroinflammation interact to promote either aEVO or AD. During younger adult life (reproduction), Aβ protofibrils function as a chaperone-like stress-response against various stressors. This confers stress-resistance for the “parental” brains, which is subsequently passed down through reproductive germ cells to offspring to confer similar stress-response capacity. While initially evolutionarily advantageous, accumulating brain AP protofibrils eventually manifest into neurodegeneration related to extended lifespan in modern times. In other words, aggregation of APs likely acts synergistically with neuroinflammation to promote protective evolutionary adaptations during reproduction for aEVO. This co-stimulatory relationship during aging ultimately contributes to AD through antagonistic pleiotropy. Accordingly, therapies countering Aβ aggregation simultaneously combined with anti-inflammatory agents might prove exponentially more effective than Aβ immunotherapies alone.

Of relevance, we thus proposed the hypothetical concept of amyloidogenic evolvability (aEVO), which is an AP protofibril-induced adaptation against diverse bio-stressors, including oxidative stress and inflammation (refer to Figure 2) (Hashimoto et al., 2018a; 2018b). Notwithstanding extensive investigation into the role of APs relevant to neurodegeneration, the precise biological functions of APs have remained elusive. Regarding this, we proposed that evolvability might be a physiological function of APs. The aEVO concept originated from the evolvability of yeast prion which is essential for yeast to survive under stressful conditions (Tyedmers et al., 2008). Similarly, in human brain, neurons are also confronted by a diverse array of environmental biostressors. Also, like yeast prion, APs are unfolded proteins with intrinsically disordered structure, which are then prone to aggregate to form protofibrils (Uversky, 2014). We hypothesized that AP protofibrils, in a sense, might act as a stress-response molecule, and could be produced in response to diverse stressors, conferring stress-resistance for the adult “parental” brain, followed by vertical transmission of protofibrils to offspring *via* germ cells in a prion-like fashion. Notably, APs were previously shown to be abundant in semen and ovarian fluids (Roan et al., 2017, National Toxicology Program. 2017). The structurally-encoded information within AP protofibrils then provides an offspring’s brain with stress protection, allowing much better resistance against forthcoming biological stressors. Although aEVO is evolutionarily beneficial, as AP protofibrils gradually accumulate, AD eventually manifests through antagonistic pleiotropy, a well-established aging theory by Williams. On this basis, AP aggregation and neuroinflammation likely interact to promote beneficial aEVO during reproduction, which, as a co-stimulatory relationship during aging, leads to AD pathogenesis through antagonistic pleiotropy.

## Discussion

Although recent multiple phase 3 clinical studies of the passive immunotherapies using anti-Aβ monoclonal antibodies including aducanumab and lecanamab were shown to delay the progression of cognitive decline in AD (Budd Haeberlein et al., 2022; Van Dyck et al., 2023), there remains uncertainty whether the current anti-Aβ immunotherapies are sufficiently efficacious to adequately treat AD. In the present paper, we suggest that Aβ aggregation and innate neuroimmunity are mutually interactive in normal neurosynaptic function, learning and memory, such that inflammatory dysregulation during aging, which causes chronic progressive neuroinflammation, then might predispose the human brain to AD neurodegeneration. Also, theoretically, this interaction promotes aEVO during earlier reproductive life, then manifesting as AD during aging through antagonistic pleiotropy. Considering this, we predict that to be effective, therapies against AD and neurodegeneration should be directed toward a combination of both anti-aggregational agents simultaneously with selective and novel anti-inflammatory strategies. With regard to the latter, several unique possibilities should be considered, including perhaps anti-HMGB1 antibody (Fujita et al., 2016) or specific HMGB1 inhibitors such as ethyl pyruvate and glycyrrhizic acid (Gaikwad et al., 2021; Gaikwad and Kayed, 2022) to limit TLR2/4 and RAGE signaling axes. Also, by restoring type 2 signaling through therapeutic injection of IL-33, IL-4 or IL-13, or augmenting hippocampal IL-4 with phosphatidyl serine (Nolan et al., 2005), neurosynaptic function might be preventatively enhanced or bolstered in mild cognitive impairment or in early AD. Collectively, these provocative therapeutic ideas may dramatically supplement current anti-Aβ immunotherapies as to improve overall treatment efficacy. Certainly, further investigations are required to clarify many of these complex immune relationships in aging and AD, prior to designing next-generation disease modifying drugs for AD and aging-associated disorders.

## Figures and Tables

**Table:** 

**A**	astrocytes
**AP-1**	activator protein 1
**BBB**	blood-brain barrier
**CaMKKbeta**	calcium calmodulin-dependent protein kinase kinase-β-AMP-activated protein kinase
**CCL2**	chemokine C-C motif ligand 2 (DAP12 DNAX-activation protein 12
**GPCR**	G-protein-coupled receptor
**hCSIF**	human cytokine synthesis inhibitory factor
**HMGB1**	high mobility group box 1 protein
**HPA**	hypothalamo-pituitary-adrenal axis
**IL**	interleukin
**IL-1RAcP**	IL-1 receptor accessory protein
**IFN-γ**	interferon-γ
**JAK/STAT**	Janus kinase/Signal transducer and activator of transcription
**Leuk**	leukocytes
**Mϕ**	macrophages
**MAPK**	mitogen-activated protein kinase
**M**	microglia
**MS**	multiple sclerosis
**N**	neurons
**NFκB**	nuclear factor kappa-light-chain-enhancer of activated B cells
**O**	oligodendrocytes
**PD**	Parkinson’s disease
**PI3K**	phosphatidylinositol-3-kinase
**PKC**	protein kinase C
**RAGE**	receptor for advanced glycation end-products
**SYK**	spleen tyrosine kinase
**TBI**	traumatic brain injury
**TLR**	Toll-like receptor
**TNFR**	TNF-α receptor
**TYK**	tyrosine kinase
